# *Vibrio cholerae* O1 classical and El Tor toxin-coregulated pilus subunit A (TcpA) epitopes that induce biotype-specific and cross-protective antibodies against bacterial adherence

**DOI:** 10.1128/aem.00350-26

**Published:** 2026-04-30

**Authors:** Sai Simha Reddy Vakamalla, David A. Sack, Weiping Zhang

**Affiliations:** 1 Department of Pathobiology, University of Illinois Urbana-Champaign 14589, Urbana, Illinois, USA; 2 Department of International Health, Johns Hopkins University Bloomberg School of Public Health 25802, Baltimore, Maryland, USA; Centers for Disease Control and Prevention, Atlanta, Georgia, USA

**Keywords:** *Vibrio cholerae*, cholera, TcpA, epitope, vaccine, biotype-specific immunity, biogenesis

## Abstract

The persistent and deadly cholera remains a significant burden on global health. Current countermeasures are inadequate against cholera. Toxin-coregulated pilin (TCP) has long been a target for understanding the biogenesis of *Vibrio cholerae* infection and for developing subunit vaccines against cholera. In this study, we examined TcpA B-cell epitopes of the O1 classical and El Tor biotypes, as well as the O139 serogroup, for functionality, potential biogenesis, and vaccine application. By empirically mapping TcpA functional epitopes across *V. cholerae* biotypes and serogroups, we found that antibodies raised against a shared epitope (EP #1, IQSQNMTKAAQ) provided partial protection against *V. cholerae* bacterial adherence *in vitro*. Data also showed that classical biotype TcpA epitope #6, ASKNLDLTNITHVE, is more effective in inducing functional antibodies against adherence of the classical biotype, but not against the El Tor biotype. Similarly, El Tor biotype TcpA epitopes #4 (FAAVADLGDFETSV) and #6 (GSANLNLTNITHVE) are effective in inducing functional antibodies against the adherence of El Tor biotype and O139 serogroup, but not against the classical biotype. This decodes early observations of a lack of cross-protection from TcpA-derived immunity. Interestingly, combined immunity raised against the classical epitope #6 and the El Tor epitope #6 (or #4) effectively protected against adherence from both biotypes. These results reveal the mechanism underlying the lack of protective immunity conferred by biotype-specific TcpA and indicate a combined epitope immunity for protection across biotypes and serogroups, providing a better understanding of TcpA immunity, perhaps its biogenesis, and guiding the development of cholera vaccines.

**IMPORTANCE:**

Current countermeasures are inadequate against cholera, a disease that remains a significant global health burden. Oral cholera vaccines (OCVs) prequalified by the World Health Organization (WHO) are serogroup-specific. In addition to the supply shortage, these whole-cell vaccines provide limited protection to children under 5 years of age in endemic countries, the most vulnerable population to cholera, urgently calling for more effective prevention strategies. By examining functional immunity raised against shared and biotype-specific TcpA epitopes, this study revealed the mechanism underlying why TCP-related immunity from the O1 classical biotype does not extend protection to the O1 El Tor biotype. The current study also showed that a combined biotype-epitope immunity provides cross-protection against O1 El Tor and classical biotypes, as well as against the O139 serogroup, potentially paving the way for the development of a cross-protective vaccine against *Vibrio cholerae* infection.

## INTRODUCTION

*Vibrio cholerae* is the causative agent of cholera, an ancient disease and a deadly and persistent threat to public health. *V. cholerae* infection is responsible for 1.3–4 million clinical cases and 21,000–143,000 deaths each year (1). Cholera originated in the Ganges Delta area of Bangladesh and India and, through various trade routes, spread to other regions in Asia, Europe, and the New World in the 1800s (2), then to Africa (1970) (3), Latin America (4), Hispaniola (2010) (5, 6), and other parts of Asia and the Middle East (2020s) (7, 8). The current surge in cholera outbreaks, reported by the World Health Organization (WHO), affected even countries that have remained cholera-free for years, with at least 30 countries reporting outbreaks and an additional 20 countries at risk, leaving over 1 billion people at risk of cholera (7). Currently, there are no countermeasures to effectively prevent or control cholera, particularly for young children in endemic countries.

Vaccines can significantly reduce cholera transmission and outbreaks (9, 10). Current cholera vaccines, however, are inadequate for children under 5 years in endemic countries or regions, particularly in South and Southeast Asia and Sub-Saharan Africa. The killed whole-cell oral cholera vaccines prequalified by WHO, including Dukoral, Euvichol/Euvichol-Plus, and Shanchol (which is no longer in production), mainly target *V. cholerae* O1 serogroup (10, 11), and they provide an efficacy of around 65% in adults and offer some herd protection when administered to a high proportion of the population (9, 12). However, these vaccines provided poor protection against children under 5 years of age in endemic countries and regions (13). New vaccine candidates, including the killed HillChol (14, 15), the live attenuated vaccine PanChol (16), and the conjugate vaccine CCV (17), have been shown to be safe and immunogenic, but efficacy has yet to be demonstrated in field trials. Additionally, current vaccine production capacity falls significantly short of global demand (10, 11). The supply shortage has forced the use of a single dose for outbreak control, but it is not available for preventive campaigns. Unfortunately, a single-dose regimen provides very short-term protection in adults and essentially no protection in children aged <5 years (13).

In an attempt to develop a broadly protective protein-based multivalent cholera vaccine, we applied an epitope- and structure-based vaccinology platform called multiepitope fusion antigen (MEFA) (18, 19), constructed a chimeric polyvalent protein antigen, termed as cholera MEFA, by presenting *in silico*-predicted B-cell epitopes from several virulence factors among *V. cholerae* serogroups on a backbone protein immunogen, and demonstrated that such a cholera MEFA protein antigen is broadly immunogenic and induces cross-protective antibodies against O1, O139, and non-O1/non-O139 bacterial adherence, intestinal colonization, and clinical cholera or diarrhea preclinically (20). However, immunodominant epitopes *in silico*-predicted are not necessarily functional or top-ranked functionally. To further improve the cholera MEFA protein for protective immunity, ideally, we would use functional or protective epitopes empirically identified from the key virulence determinants of *V. cholerae* to construct an optimal cholera MEFA.

Toxin-coregulated pilus (TCP), a type IV pilus composed of a 20.5-kDa major subunit or pilin TcpA, plays an essential role in *V. cholerae* intestinal colonization and onset of clinical cholera (21–24). Moreover, antibodies against TcpA (or TCP) have been shown to protect against *V. cholerae* colonization and infection (25–29), making TcpA a prominent antigen target for cholera vaccine development (29–32). However, TcpA is heterogeneous between the O1 classical and El Tor biotypes, and among different serogroups, and exhibits biotype- or serogroup-specific alleles (33–35). Antibodies to TcpA of the O1 classical biotype protect against infection from the classical biotype but do not extend to protection against the El Tor biotype (29, 36, 37). While the protective antigenic domain was narrowed down to the variable C-terminus (29), the TcpA protective B-cell epitopes, especially from the El Tor biotype, and the mechanism underlying the lack of protection across the two biotypes or serogroups conferred by TCP antibodies have not been fully revealed (28–30).

In this study, we first identified B-cell epitopes from *V. cholerae* O1 classical and El Tor biotypes, individually fused each epitope to a carrier, and immunized mice with each epitope fusion. We then examined antibodies raised against shared or biotype-specific epitopes for their roles in bacterial attachment of *V. cholerae* O1 classical and El Tor biotypes, as well as an O139 strain. We also correlated TcpA epitope heterogeneity of the O1 classical and El Tor biotypes with TcpA biotype-specific protective immunity and explored the potential application of TcpA epitopes in multivalent cholera vaccine development.

## RESULTS

### *V. cholerae* TcpA has one conserved epitope and five biotype-specific B-cell epitopes between O1 classical and El Tor biotypes

**Fig 1:**
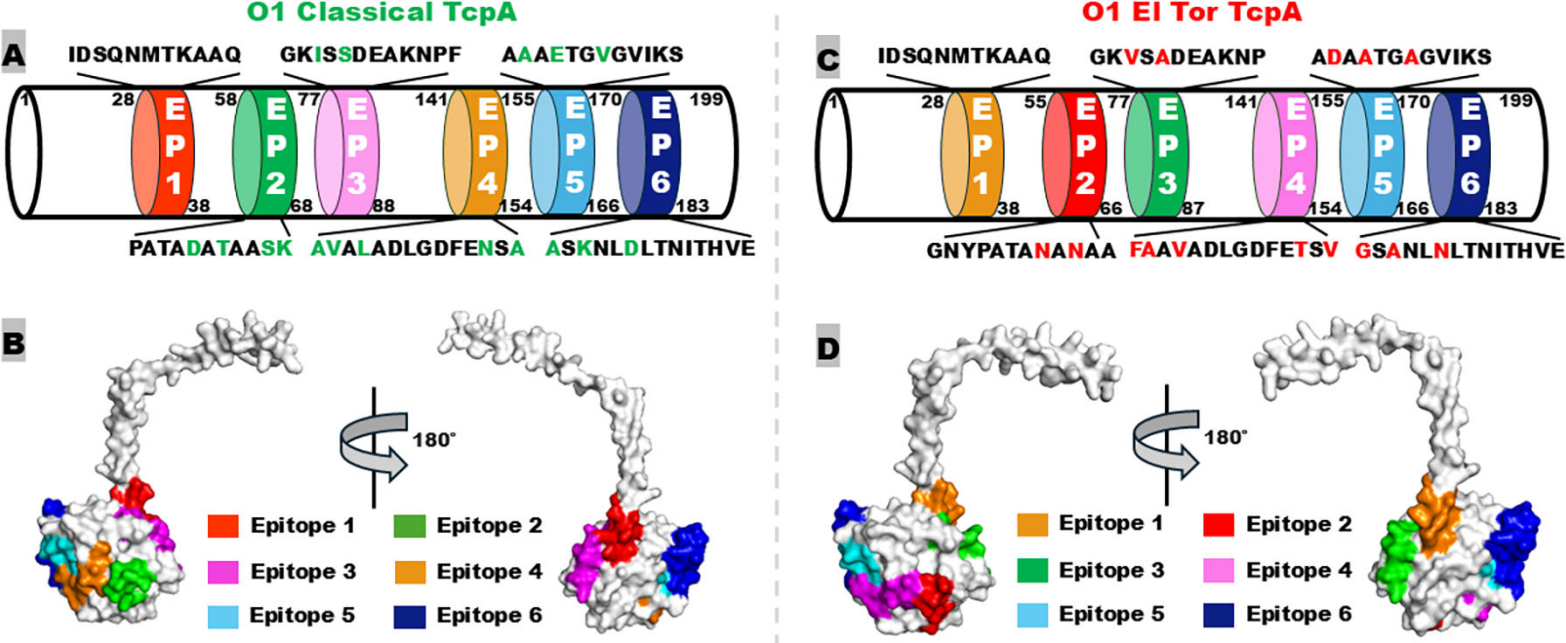
Schematic illustration of toxin-coregulated pilin (TcpA) B-cell epitopes of *Vibrio cholerae* O1 classical and El Tor biotypes. (**A**) A diagram shows the amino acid sequences and locations of *in silico*-predicted epitopes for O1 classical TcpA (strain O395; GenBank accession no. CP000627.1). Biotype-specific amino acid residues are in green. (**B**) The classical (O395) TcpA protein model (UniProt: A0A0H3AHY1) and the epitopes (in different colors), generated by Phyre 2.0. (**C**) A diagram shows epitope sequences and locations of O1 El Tor TcpA (strain N16961; GenBank accession no. AF325734.1). Biotype-specific amino acid residues are in red. (**D**) The El Tor (N16961) TcpA protein model (UniProt: Q60153) and the presence of epitopes (in different colors).

After *in silico* analysis of the *V. cholerae* TcpA sequence of the clinically relevant O1 biotypes classical and El Tor, O139, and some non-O1/non-O139 isolates (34, 35), we identified six B-cell immunodominant epitopes, EP1 to EP6 (Fig. 1). EP1 (epitope #1, TcpA_28–38_), IDSQNMTKAAQ, is shared among the two O1 biotypes and the other serogroup strains that represent different phylogenetic clades (34). The EP2 (TcpA_58–68_ of classical and TcpA_55–66_ of El Tor), EP3 (TcpA_77–88_ of classical and TcpA_77–87_ in El Tor), EP4 (TcpA_141–154_), EP5 (TcpA_155–166_), and EP6 (TcpA_170–183_) varied at two to five amino acid residues in the classical biotype from the El Tor biotype (Fig. 1). Additional variations were found in these five epitopes among non-O1/non-O139 strains (34, 35).

### TcpA epitope fusions induced a robust specific antibody response in mice

**Fig 2:**
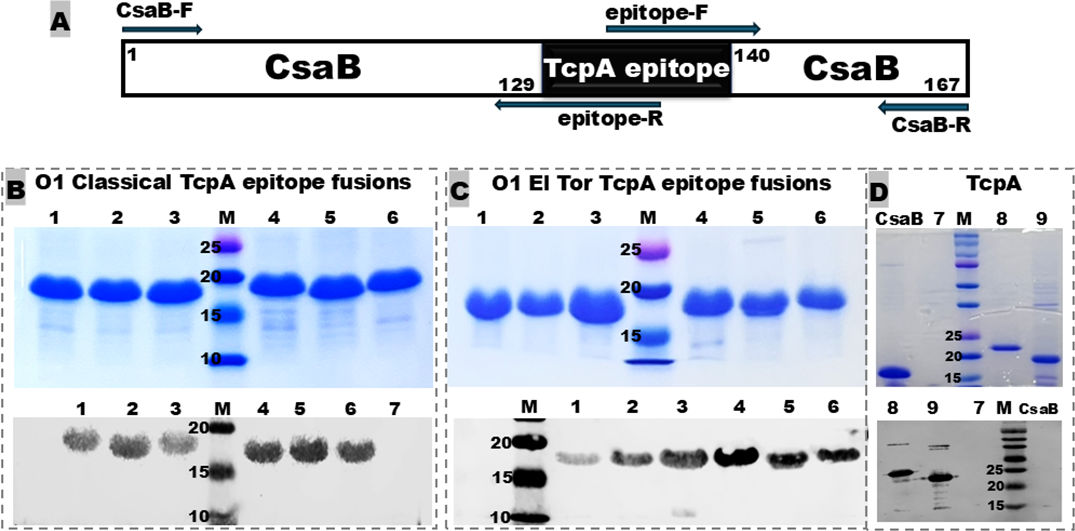
Schematic illustration and characterization of *Vibrio cholerae* O1 classical and El Tor TcpA epitope fusion proteins. (**A**) A diagram shows the fusion of a *V. cholerae* TcpA epitope to the carrier protein CsaB (the major structural subunit of the adhesin CS4 of enterotoxigenic *E. coli*) using overlapping extension with specifically designed PCR primers. (**B**) SDS-PAGE Coomassie blue staining and Western blot of six classical biotype-specific TcpA epitope fusion proteins. 1 to 6, EP1 to EP6; 7, *E. coli* BL21 host cell proteins as a negative control. (**C**) SDS-PAGE Coomassie blue staining and Western blot of six El Tor biotype-specific TcpA epitope fusion proteins. 1 to 6, EP1 to EP6. (**D**) SDS-PAGE Coomassie blue staining and Western blot (with anti-TcpA polyclonal antibodies) of the protein carrier (CsaB), two biotype-specific TcpA proteins, and *E. coli* BL21 host proteins. 7, host proteins of *E. coli* BL21; 8, 6×His-tagged classical TcpA (22.8 kDa); 9, El Tor TcpA protein (20.5 kDa); CsaB, carrier CsaB (17.4 kDa), a major subunit of enterotoxigenic *E. coli* adhesin CS4; and M, molecular marker (in kDa).

Each TcpA epitope was inserted into a heterologous protein carrier, CsaB, via epitope substitution (Fig. 2A). CsaB is the major subunit of enterotoxigenic *Escherichia coli* (ETEC) adhesin CS4, and it does not react with anti-TcpA polyclonal antisera (nor reactivity of anti-CsaB antibodies with TcpA recombinant proteins). Each recombinant epitope fusion protein was expressed at a size of about 17.6 kDa and a purity of over 95%, and each biotype-specific epitope fusion protein was recognized by mouse antisera specific to classical TcpA (Fig. 2B) or El Tor TcpA (Fig. 2C). All TcpA epitopes remained antigenic (*in silico*) after being fused to CsaB carrier. Additionally, full-length 6×His-tagged classical TcpA (22.8 kDa) and tag-less El Tor TcpA (20.5 kDa; gene carries nucleotides of NheI restriction site, thus cloned at NcoI site) were expressed as positive-control immunogens. Both TcpA recombinant proteins, but not the carrier protein CsaB or total proteins of the host *E. coli* BL21, reacted with anti-TcpA antisera (Fig. 2D).

**Fig 3:**
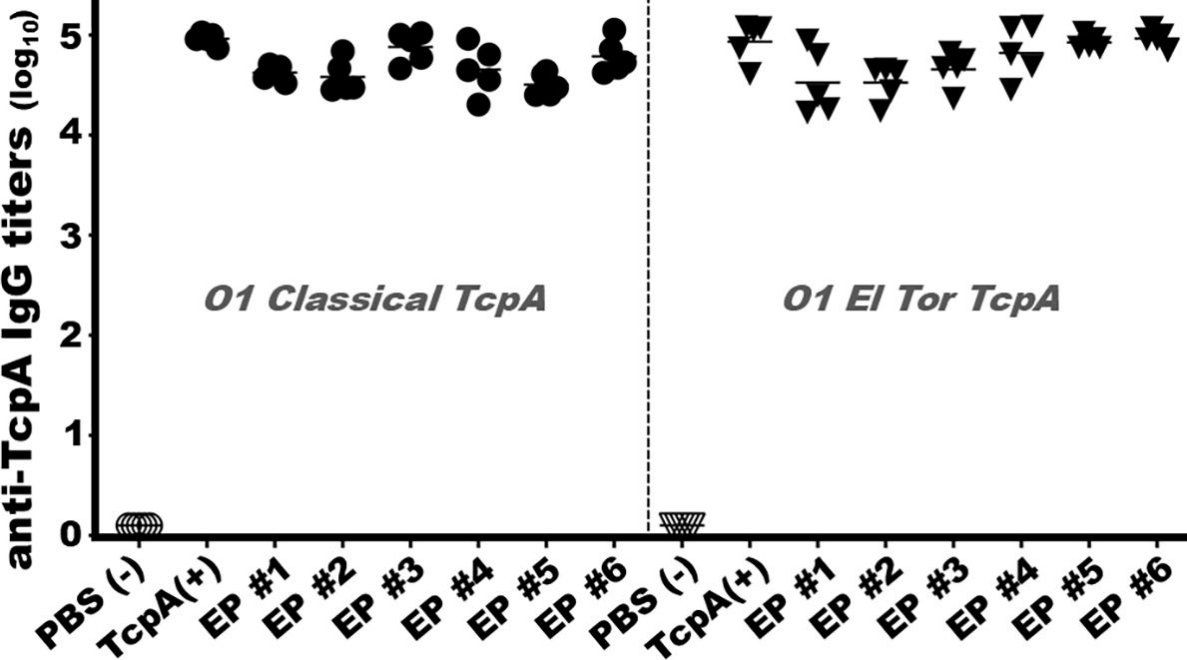
Mouse serum IgG titers (log_10_) to *V. cholerae* O1 classical or El Tor TcpA after intramuscular immunization (three doses, 2-week interval) with each biotype-specific TcpA epitope fusion protein. Recombinant full-length classical or El Tor TcpA protein was used as the coating antigen in enzyme-linked immunosorbent assays. EP represents each CsaB-TcpA epitope fusion protein. Sera from mice immunized with recombinant TcpA were used as the positive control (+), and sera from mice injected with PBS were used as the negative control (−). Each dot represents a titer from an individual mouse.

Mice intramuscularly immunized with each TcpA epitope fusion protein developed a robust antibody response to the classical or El Tor TcpA (Fig. 3). Mouse serum IgG titers derived from the conserved EP1 epitope were 4.6 ± 0.08 and 4.5 ± 0.33 (log_10_), respectively, to the classical or El Tor TcpA. Serum IgG titers from classical EP2, EP3, EP4, EP5, or EP6 fusion were 4.6 ± 0.17, 4.9 ± 0.16, 4.7 ± 0.25, 4.5 ± 0.11, and 4.8 ± 0.17, respectively. Serum IgG titers from the mice immunized with the full-length classical biotype-specific TcpA recombinant protein were 5.0 ± 0.06.

Serum IgG titers from the El Tor biotype-specific EP2, EP3, EP4, EP5, or EP6 were 4.5 ± 0.18, 4.7 ± 0.18, 4.8 ± 0.27, 4.9 ± 0.07, and 5.0 ± 0.08, respectively. Serum IgG titers from the mice immunized with the full-length El Tor TcpA were 4.9 ± 0.20.

No IgG responses to either recombinant TcpA protein were detected in the control mice (injected with PBS).

### Classical biotype TcpA epitope #6 and El Tor biotype epitopes #4 and #6 were the most effective at inducing functional antibodies against *V. cholerae in vitro* attachment

**Fig 4:**
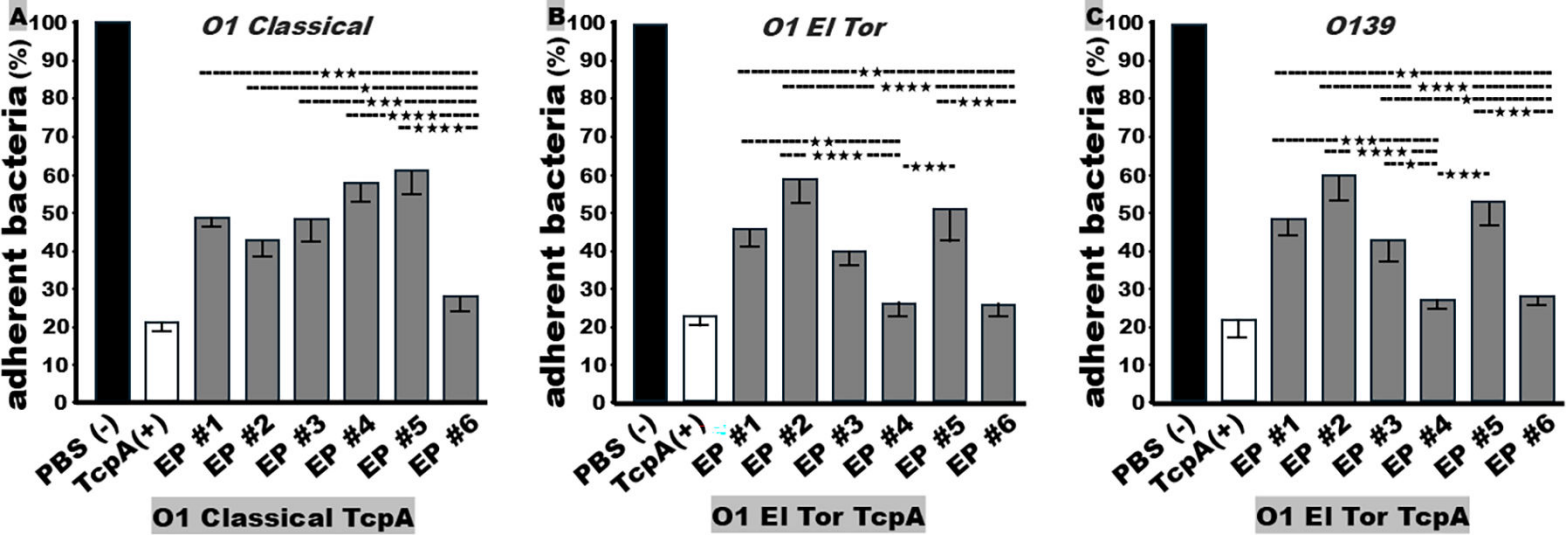
Mouse serum antibody inhibition against adherence (or attachment) of *V. cholerae* O1 classical (O395), El Tor (N16961), or O139 (Bengal) to Caco-2 cells. (**A**) Adherent *V. cholerae* O1 classical O395 bacteria (CFUs in percentage) to Caco-2 cells after incubation with heat-inactivated sera from mice immunized with each classical biotype-specific TcpA epitope fusion (EP1 to EP6), PBS (−), or classical biotype TcpA (+). (**B**) Adherent *V. cholerae* O1 El Tor N16961 bacteria (CFUs in percentage) to Caco-2 cells after incubation with heat-inactivated sera from mice immunized with each El Tor biotype-specific TcpA epitope fusion (EP1 to EP6), PBS (−), or El Tor biotype TcpA (+). (**C**) Adherent *V. cholerae* O139 Bengal bacteria (CFUs in percentage) to Caco-2 cells after incubation with heat-inactivated sera from mice immunized with each El Tor biotype-specific TcpA epitope fusion (EP1 to EP6), PBS (−), or El Tor biotype TcpA (+). ★, *P* < 0.05; ★★, *P* < 0.01; ★★★, *P* < 0.001; and ★★★★, *P* < 0.0001.

Mouse serum antibodies derived from each TcpA epitope fusion reduced *in vitro* bacterial attachment (or adherence) of O1 classical, O1 El Tor, and an O139 strain (Fig. 4). After incubation with serum from mice immunized with classical biotype TcpA epitope fusion EP1, EP2, EP3, EP4, EP5, or EP6, *V. cholerae* classical biotype strain O395 showed a reduction in attachment to Caco-2 cells by 51%, 57%, 52%, 42%, 39%, or 72%, respectively, compared to bacteria treated with control mouse sera. The adherence inhibition against the classical biotype from antibodies derived from EP6 was significantly better than EP1 (*P* < 0.001), EP2 (*P* < 0.05), EP3 (*P* < 0.001), EP4 (*P* < 0.0001), or EP5 (*P* < 0.0001), but was not different compared to antibodies raised against the full-length classical TcpA (TcpA_1–199_), which reduced the O395 bacterial adherence by 78% (Fig. 4A).

El Tor biotype strain N16961, after incubation with sera from mice immunized with the El Tor biotype-specific TcpA epitopes EP1, EP2, EP3, EP4, EP5, or EP6, reduced bacterial adherence to Caco-2 cells by 53%, 40%, 59%, 73%, 48%, or 73%, respectively (Fig. 4B). Antibodies derived from EP4 or EP6 were significantly more effective than EP1 (*P* < 0.01), EP2 (*P* < 0.0001), or EP5 (*P* < 0.001) in adherence inhibition against N16961, but not than EP3 (*P* = 0.05) or the full-length TcpA_1-199_. Serum antibodies raised against El Tor TcpA_1-199_ reduced the N16961 bacterial adherence by 76%.

Mouse serum antibodies raised against El Tor biotype-specific EP4 and EP6 were also the most effective against adherence of O139 Bengal strain (Fig. 4C). The adherence to Caco-2 cells by this Bengal strain was reduced by 50%, 39%, 57%, 72%, 47%, and 71%, respectively, after incubation with sera from the mice immunized with EP1, EP2, EP3, EP4, EP5, or EP6 fusion protein. Adherence inhibition activity from antibodies derived from EP4 and EP6 was significantly more effective than antibodies from EP1 (*P* < 0.01), EP2 (*P* < 0.0001), EP3 (*P* < 0.05), or EP5 (*P* < 0.001), but not the full-length TcpA_1-199_, which reduced Bengal strain adherence by 77%.

### A mixture of serum antibodies raised from a classical TcpA epitope and an El Tor epitope provided cross-biotype protection against both O1 serotypes

**Fig 5:**
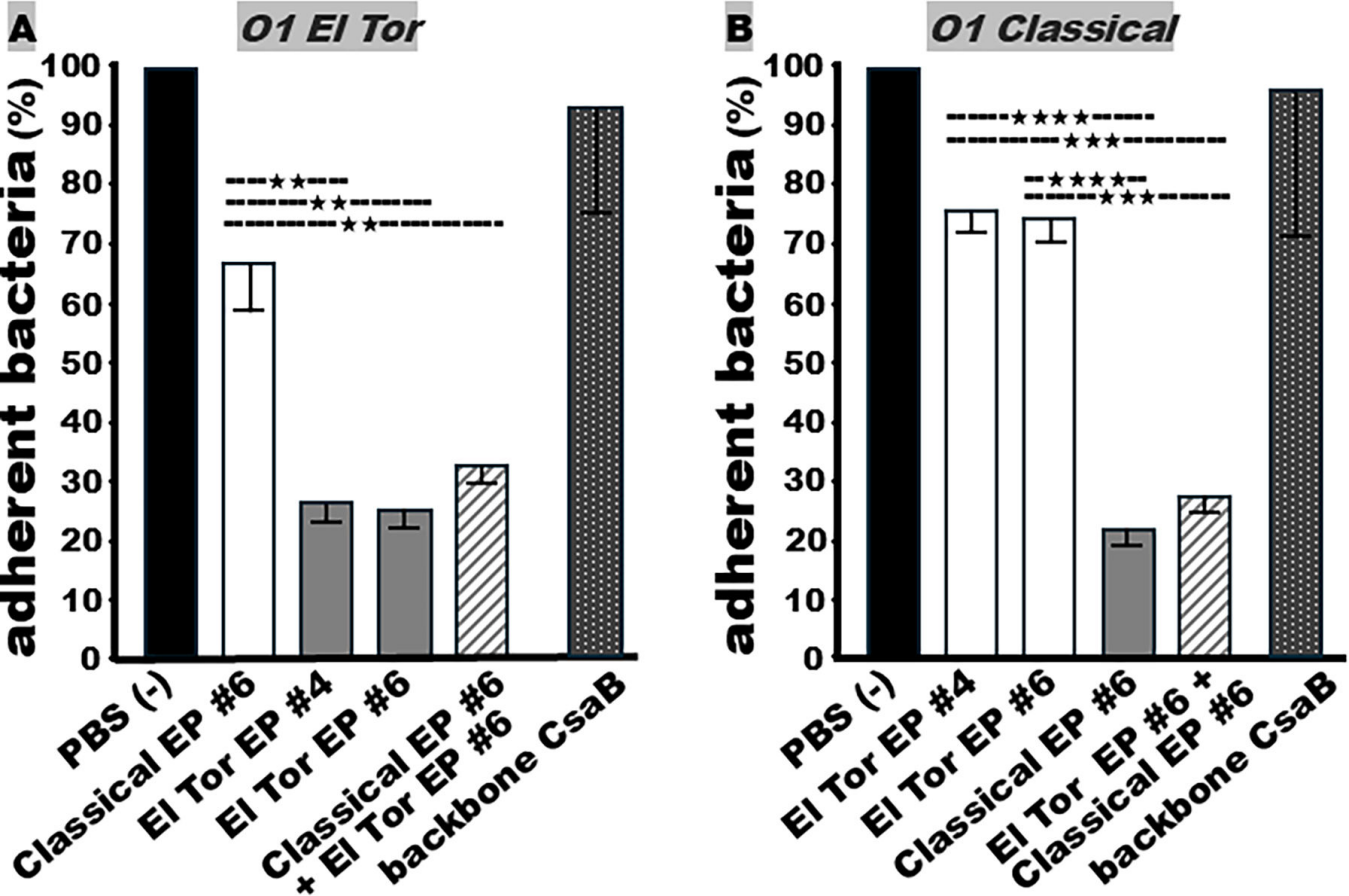
Antibody adherence inhibition assays to compare biotype-specific or mixed serum antibodies against *V. cholerae* O1 classical and El Tor bacterial adherence. (**A**) O1 El Tor N16961 bacteria (CFUs in percentage) adherent to Caco-2 cells after incubation with heat-inactivated sera from mice immunized with classical biotype-specific TcpA epitope fusion EP6, sera from mice immunized with El Tor biotype-specific TcpA epitope fusion EP4 or EP6, or a mixture of sera from the mice immunized with classical epitope EP6 with the sera from the mice immunized with El Tor-specific epitope fusion EP6. (**B**) O1 classical O395 bacteria (CFUs in percentage) adherent to Caco-2 cells after incubation with sera from mice immunized with El Tor-specific TcpA epitope fusion EP4 or EP6, sera from the mice immunized with classical-specific TcpA epitope fusion EP6, or a mixture of sera from the mice immunized with classical epitope EP6 with the sera from the mice immunized with El Tor-specific epitope fusion EP6. Sera from mice immunized with carrier CsaB (backbone CsaB) or PBS (−) as the control. ★★, *P* < 0.01; ★★★, *P* < 0.001; and ★★★★, *P* < 0.0001.

Mouse serum antibodies raised against the TcpA epitopes showed biotype specificity against *V. cholerae* adherence or attachment. Mouse serum antibodies from classical TcpA EP6, which were most effective against classical O395 bacterial adherence, were significantly less effective against adherence of El Tor biotype N16961 (Fig. 5A). Similarly, the El Tor TcpA EP4- or EP6-induced mouse serum antibodies, which were most effective against adherence of O1 El Tor biotype or O139 Bengal strain, were not effective against adherence of the O1 classical biotype strain (Fig. 5B).

However, a mixture of sera from the mice immunized with the classical biotype EP6 and sera raised from the El Tor biotype EP6 or EP4 protected against adherence of both biotypes (Fig. 5). Mouse sera raised against the classical EP6 epitope fusion, when combined with sera from mice immunized with El Tor EP6 or EP4 epitope fusion, were equally effective against the adherence of El Tor strain N16961 as sera from mice immunized with El Tor EP4 or EP6 fusion (Fig. 5A). Similarly, the mixed mouse sera antibodies were also equally effective against adherence of classical strain O395 as sera raised against the classical EP6 fusion (Fig. 5B).

Mouse sera raised against carrier protein CsaB were not effective against the adherence of the N16961 or O395 strain.

## DISCUSSION

While oral rehydration therapy and improvements in drinking water, sanitation, and hygiene can prevent or treat cholera outbreaks, these interventions are not always available in many cholera-endemic countries. Vaccines are currently considered a more practical and effective countermeasure. Given its critical role in *V. cholerae* infection and its potential application in cholera subunit vaccine development, the TcpA pilin remains a target of interest for understanding its molecular biogenesis and immunological properties. By comparing TcpA epitopes of O1 classical and El Tor biotypes for functional immunity in this study, we found that antibodies targeting the conserved epitope (EP1, TcpA_28–38_) reduced bacterial adherence by only 51% in O1 classical strain O395, 53% in El Tor strain N16961, and 51% in O139 strain Bengal. In contrast, the biotype-specific epitopes induced better functional immunity. Serum antibodies derived from the classical EP6 (TcpA_170–183_) reduced adherence of the O1 classical strain O395 by over 70%, significantly better than antibodies derived from the common epitope EP1. Similarly, serum antibodies raised against El Tor EP4 (TcpA_141–154_) or EP6 (TcpA_170–183_) were significantly more effective against O1 El Tor N16961 or O139 Bengal strain. These results confirmed that O1 TcpA immunity is specific to its biotype, as observed in both field exposure and experimental studies (29), and may also reveal mechanisms underlying the lack of cross-protection to the other biotype. This study also demonstrated that combined immunity conferred by the functional epitopes of two biotypes provided cross-protection against bacterial attachment, adding information for the future development of cross-protective protein-based, particularly epitope-based, cholera vaccines.

The O1 classical biotype TcpA, particularly its C-terminal segments, has been well studied for its role in protective immunity against intestinal colonization and infection from a classical biotype strain (26, 28–30, 38, 39). Early studies indicated that synthetic peptides, including classical TcpA4 (145–168), TcpA5 (157–182), and TcpA6 (174–199), conjugated to keyhole limpet hemocyanin (KLH), elicited antibodies to TcpA or TCP. Moreover, passive antibodies raised against the TcpA4, TcpA5, or TcpA6 conjugate protected 89%, 50%, and 70% of infant mice, respectively, when infected with a lethal dose of the classical strain O395 (28). This TcpA4 (145–168) peptide overlaps with epitopes EP4_(141–154)_ and EP5_(155–166)_ identified from this study, TcpA5 (157–182) covers nearly the entire epitope EP5_(155–166)_ and epitope EP6_(170–183)_, whereas TcpA6 (174–199) contains epitope EP6_(170–183)_. Results from our *in vitro* antibody adherence inhibition assay indicated that antibodies induced by the EP6 epitope are most effective at inhibiting the adherence of the classical strain O395. Unlike the study by Sun et al. (28), antibodies raised against EP4 or EP5, which are covered by their most effective peptide TcpA4, were less effective at inhibiting O395 adherence in the current study. A subsequent study by the Taylor laboratory, using shortened peptides (conjugated to KLH), found that classical TcpA15 (170–183) was the most effective in inducing antibodies (in rabbit sera) to passively protect infant mice against challenge with the classical biotype strain O395 (29). The study also indicated that antibodies raised against TcpA11 (141–154) conjugates provided some protection against infection from strain O395 (29). TcpA11 is identical to our EP4 epitope, and TcpA15 is the same as our EP6 epitope. Results from this study confirmed that EP6 (TcpA15) is the most functional epitope for the classical biotype and indicated that the EP4 (TcpA11) fusion was the second least effective at inhibiting adherence by the classical strain O395.

Compared to the O1 classical biotype TcpA, the TcpA of the El Tor biotype has been less studied in the past, and its protective peptides or epitopes have not been identified until this study. An early study comparatively examined the immunogenicity of peptides TcpA4, TcpA5, and TcpA6 from the O1 classical and El Tor biotypes and concluded that the three peptides of the El Tor biotype were also immunogenic (40). However, the study did not examine the functionality or protective effects of antibodies conferred by these El Tor TcpA peptides. Data from the current research identified, for the first time, that both El Tor-specific TcpA EP4 and EP6 are equally effective at inducing functional antibodies against adherence from the El Tor strain N16961, providing necessary information for vaccine development with El Tor TcpA antigens, particularly given that El Tor is currently the circulating strain causing clinical cholera outbreaks. While the classical strain is no longer associated with cholera outbreaks, a study reported that hybrid strains isolated from hospital patients, classified as El Tor biotype, indeed carried the classical TcpA gene (41), reminding us of the need to include classical TcpA antigenic components in cholera vaccine development.

Interestingly, the biotype-specific functional immunity conferred by TcpA EP6 is defined by three heterogeneous amino acid residues: TcpA_170_, TcpA_172_, and TcpA_175_. The classical biotype TcpA residues K172 and D175 have been shown to play a role in maintaining TCP functions, including agglutination, intestinal colonization, phage transduction, and serum resistance (42). The alanine at position 170 in the classical biotype TcpA, however, was not included in the same study. This TcpA170 could also play a role in TCP biogenesis or in protection against *Vibrio cholerae* adherence or colonization. Future mutagenesis studies to mutate each residue within the epitope and assess the functional immunity conferred by each derived epitope against bacterial adherence and, ideally, against intestinal colonization can systematically evaluate if these three residues, individually or collectively, dictate the biotype specificity of TcpA epitope EP6.

We noted that the conserved TcpA epitope, EP1, when surface-exposed on the epitope fusion protein, was strongly immunogenic and induced antibodies that showed some activity against the *in vitro* adherence of O1 classical and El Tor biotypes, as well as an O139 strain. Reducing adherence of two O1 biotypes and O139 serogroup by EP1 immunity suggests a potential application of EP1 as an antigenic component in a cross-protective cholera vaccine, seemingly contradicting the established notion that immunity induced by TCP pili lacks cross-protection. The specific tertiary structure of EP1 on TCP is likely the cause. EP1 (TcpA_28–38_) is located in the α-helix of the N-terminus of TcpA pilin, which is believed to be tightly packed and buried at the helical-core subdomain-subdomain interface to form the bundled filamentous TCP pili (43, 44), thereby rendering TCP ineffective in inducing an EP1-specific antibody response and cross-protective immunity. If TcpA EP1 became fully exposed, it could provide some degree of cross-protection.

While a surface-exposed conserved EP1 could be a target antigen for TcpA, the most functional but biotype-specific classical EP6 and El Tor EP4 and EP6, which are surface-exposed at the epitope fusion proteins or TCP, should be our primary antigen targets (also functional epitopes from other *V. cholerae* key virulence factors) for the future development of epitope-based multivalent cholera vaccines. Although antibodies raised against the two EP6 epitopes (and El Tor EP4) are biotype-specific, a mixture of serum antibodies derived from classical EP6 and El Tor EP6 or EP4 was shown to inhibit bacterial adherence of both O1 biotypes, achieving cross-protection against different biotypes and likely serogroups through TcpA-induced immunity. Therefore, incorporating classical EP6 and El Tor EP6 (and/or EP4) into a multiepitope fusion antigen, along with functional epitopes from other virulence factors such as cholera toxin and flagellins, can lead to a broadly immunogenic and cross-protective cholera subunit vaccine.

We need to emphasize that the current study was limited to mapping the functional epitopes by examining the activities of the TcpA epitope-derived immunity against *V. cholerae* biotype and serogroup bacterial adherence *in vitro*. Future *in vivo* studies to examine epitope immunity against *V. cholerae* colonization in the small intestines, using an adult rabbit colonization model (45, 46), or against clinical cholera in the passive infant mouse model assay (29) or the passive infant rabbit protection model (20), can further define the utility of these TcpA epitopes in cholera vaccine development. We also need to point out that the identified functional TcpA epitopes will not be the sole antigens for a cholera vaccine. Rather, we will map the functional epitopes from other virulence factors, including cholera toxin, flagellins, and neuraminidase, of *V. cholerae* O1, O139, and perhaps non-O1/non-O139 serogroups. The identified top-ranked functional epitopes will be presented by a backbone protein for the construction of an epitope- and structure-based multiepitope fusion antigen. After *in vitro* (cell lines) confirmation of antibody functions against bacterial attachment, enterotoxicity, and motility, but more importantly, preclinical efficacy against intestinal colonization and clinical cholera or diarrhea in animal models, this polyvalent MEFA immunogen will be an optimal polyvalent antigen for the development of a cross-protective cholera vaccine.

In conclusion, the current study confirmed classical TcpA_170–183_ as the functional epitope against the O1 classical biotype, identified El Tor TcpA_141–154_ and TcpA_170–183_ as two functional epitopes against the O1 El Tor biotype, and demonstrated a mixed immunity from epitopes of two biotypes protective against classical and El Tor strains, providing helpful information to understand TCP biogenesis further and accelerate our efforts in developing epitope-based cross-protective subunit vaccines against cholera.

## MATERIALS AND METHODS

### Bacterial strains and plasmids

*Vibrio cholerae* strains supplied by Biodefense and Emerging Infections Research Resources Repository and recombinant *E. coli* strains expressing recombinant classical or El Tor TcpA or TcpA epitope fusion proteins used in this study are described in Table 1. The O1 classical and El Tor TcpA and TcpA epitope fusion genes were cloned into the pET28a vector (Novagen, Madison, WI, USA) and expressed in the *E. coli* BL21(DE3) strain (Agilent Technologies, Santa Clara, CA, USA).

**TABLE 1:** *Vibrio cholerae* and *E. coli* strains used in this study

Strain	Relevant characteristic(s)	Source or reference
DH5α	F^–^ ϕ80*lac*ZΔM15 Δ(*lacZYA argF*)*U169 recA1 endA1 hsdR17*(r_K_^–^ m_K_^+^) *phoA supE44* λ^–^ *thi-1 gyrA96 relA1*	Thermo Fisher
BL21(DE3)	B F^−^ *ompT hsdS*(r_B_^−^ m_B_^−^) *dcm^+^* Tet^r^ gal *λ*(DE3) *endA Hte* [*argU proL* Cam^r^]	Agilent
9765	ETEC CS4 adhesin major subunit (CsaB) in DH5α/pET28α, Kan^+^	(47)
9785	*Vibrio cholerae,* strain O395, O1, Ogawa, classical	BEI NR-9906 (ATCC 39541)
9817	*Vibrio cholerae,* strain MO45, O139 (Bengal)	BEI NR-144 (ATCC 51394)
9854	*Vibrio cholerae,* strain N16961, O1, Inaba, El Tor	BEI NR-147 (ATCC 39315)
9771	“El Tor TcpA + pET28α” in BL21(DE3)	This study
9808	“Classical TcpA + pET28α” in BL21(DE3)	This study
10047	“CsaB-Classical_TcpA_EP1 fusion + pET28α” in BL21(DE3)	This study
10048	“CsaB-Classical_TcpA_ EP2 fusion + pET28α” in BL21(DE3)	This study
10049	“CsaB-Classical_TcpA_ EP3 fusion + pET28α” in BL21(DE3)	This study
10050	“CsaB-Classical_TcpA_ EP4 fusion + pET28α” in BL21(DE3)	This study
10051	“CsaB-Classical_TcpA_ EP5 fusion + pET28α” in BL21(DE3)	This study
10052	“CsaB-Classical_TcpA_ EP6 fusion + pET28α” in BL21(DE3)	This study
10059	“CsaB-El Tor_TcpA_EP1 fusion + pET28α” in BL21(DE3)	This study
10060	“CsaB-El Tor_TcpA_EP2 fusion + pET28α” in BL21(DE3)	This study
10061	“CsaB-El Tor_TcpA_EP3 fusion + pET28α” in BL21(DE3)	This study
10062	“CsaB-El Tor_TcpA_EP4 fusion + pET28α” in BL21(DE3)	This study
10063	“CsaB-El Tor_TcpA_EP5 fusion + pET28α” in BL21(DE3)	This study
10064	“CsaB-El Tor_TcpA_EP6 fusion + pET28α” in BL21(DE3)	This study

### *Vibrio cholerae* TcpA B-cell epitope *in silico* identification and epitope fusion construction

Computer-based *in silico* prediction programs available through publicly accessible bioinformatics tools were used to identify immunodominant B-cell epitopes of TcpA from *V. cholerae* O1 classical and El Tor biotypes. TcpA epitope homology, especially among O1 classical and El Tor biotypes, was examined. The TcpA nucleotide or amino acid sequences (or Protein Data Bank identifiers) of classical biotype strain O395 and El Tor biotype strain N16961 were submitted to BepiPred 2.0 (https://services.healthtech.dtu.dk/service.php?BepiPred-2.0/) and the IEDB B-cell epitope prediction tool (http://tools.iedb.org/bcell). Epitopes were selected based on antigenicity scores from various algorithms and the Parker Index of Hydrophilicity. Using this multi-criteria analysis, we identified linear B-cell epitopes of TcpA for both biotypes. To assess surface positioning/exposure, we mapped these candidate epitopes onto predicted 3-D structural models of the TcpA proteins using PyMOL version 2.3.2 (Schrödinger, LLC; http://www.pymol.org) and structural predictions from Phyre2 (http://www.sbg.bio.ic.ac.uk/~phyre2/html/page.cgi?id=index). Only surface-accessible epitopes were included in consideration of fusion and downstream expression.

To identify a proper carrier for TcpA epitope fusion preparation, we evaluated a few candidates, including maltose-binding protein, chicken ovalbumin, enterotoxigenic *E. coli* adhesin CFA/I major subunit CfaB, and ETEC adhesin CS4 subunit CsaB. The carrier should not react with antibodies raised against TcpA, nor should antibodies raised against the carrier react with TcpA. Given no specific cross-reactivity among the examined carrier proteins, we selected CsaB (6×His-tagged; 17.4 kDa) for its small size, strong immunogenicity, structural stability, and reliable expression and purification.

CsaB-TcpA epitope fusions were constructed by substituting a CsaB epitope with each TcpA epitope using splicing by overlap extension PCR, with specific PCR primers (Table 2), as described previously (48–50). To insert a TcpA epitope into the CsaB carrier, we overlapped two PCR products amplified, respectively, with CsaB-F/TcpA-EP-R and TcpA-EP-F/CsaB-R, as illustrated in Fig. 2. The overlapped product was digested with the NheI and EagI restriction enzymes and cloned into the vector pET28α, confirmed for staying in the correct reading frame with Sanger sequencing, and then expressed by the *E. coli* BL21 (DE3) strain.

**TABLE 2:** Primers used in this study to amplify the CsaB carrier, full-length TcpA, and TcpA epitope fusion genes

Primer	Sequence (5′→3′)^*a*^	Amplified region
CsaB-F	CGGGCTAGCGTAGAGAAAAATATCACTGTA	Forward primer to amplify *E. coli CsaB* gene, with NheI site
CsaB-R	TTACGGCCGTTATTATGATGCTAAGGTCATTAAGATAGA	Reverse primer to amplify *E. coli CsaB* gene, with EagI site
Classical_TcpA_EP1-F	CAGAATATGACCAAGGCCGCGCAAttagtgattggtgcgactacagca	Paired with CsaB-R to insert the classical TcpA EP1 epitope into CsaB
Classical_TcpA_EP1-R	GGCCTTGGTCATATTCTGCGAATCAATtgacgttccaaaatttaattc	Paired with CsaB-F to insert the classical TcpA EP1 epitope into CsaB
Classical_TcpA_EP2-F	GCTGATGCGACAGCTGCTAGTAAGgtgattggtgcgactacagcacaa	Paired with CsaB-R to insert the classical TcpA EP2 epitope into CsaB
Classical_TcpA_EP2-R	AGCAGCTGTCGCATCAGCTGTTGCTGGtgacgttccaaaatttaattc	Paired with CsaB-F to insert the classical TcpA EP2 epitope into CsaB
Classical_TcpA_EP3-F	TCATCCGATGAGGCAAAAAACCCATTCattggtgcgactacagcacaa	Paired with CsaB-R to insert the classical TcpA EP3 epitope into CsaB
Classical_TcpA_EP3-R	TTTTGCCTCATCGGATGATATTTTACCtgacgttccaaaatttaattc	Paired with CsaB-F to insert the classical TcpA EP3 epitope into CsaB
Classical_TcpA_EP4-F	GCAGATCTAGGTGATTTTGAGAATTCTGCAgcgactacagcacaagcacaa	Paired with CsaB-R to insert the classical TcpA EP4 epitope into CsaB
Classical_TcpA_EP4-R	AAAATCACCTAGATCTGCAAGTGCTACTGCtgacgttccaaaatttaattc	Paired with CsaB-F to insert the classical TcpA EP4 epitope into CsaB
Classical_TcpA_EP5-F	GAGACAGGCGTTGGTGTGATCAAATCTattggtgcgactacagcacaa	Paired with CsaB-R to insert the classical TcpA EP5 epitope into CsaB
Classical_TcpA_EP5-R	CACACCAACGCCTGTCTCAGCCGCTGCtgacgttccaaaatttaattc	Paired with CsaB-F to insert the classical TcpA EP5 epitope into CsaB
Classical_TcpA_EP6-F	GATCTAACGAACATCACTCACGTTGAGgcgactacagcacaagcaccaacggcg	Paired with CsaB-R to insert the classical TcpA EP6 epitope into CsaB
Classical_TcpA_EP6-R	AGTGATGTTCGTTAGATCTAAATTCTTACTAGCtgacgttccaaaatttaattc	Paired with CsaB-F to insert the classical TcpA EP6 epitope into CsaB
El Tor_TcpA_EP1-F	CAGAATATGACTAAGGCTGCGCAAttagtgattggtgcgactacagca	Paired with CsaB-R to insert the El Tor TcpA EP1 epitope into CsaB
El Tor_TcpA_EP1-R	AGCCTTAGTCATATTCTGCGAATCAATtgacgttccaaaatttaattc	Paired with CsaB-F to insert the El Tor TcpA EP1 epitope into CsaB
El Tor_TcpA_EP2-F	CCAGCTACCGCAAACGCAAATGCTGCTattggtgcgactacagcacaa	Paired with CsaB-R to insert the El Tor TcpA EP2 epitope into CsaB
El Tor_TcpA_EP2-R	TGCGTTTGCGGTAGCTGGATAATTACCtgacgttccaaaatttaattc	Paired with CsaB-F to insert the El Tor TcpA EP2 epitope into CsaB
El Tor_TcpA_EP3-F	TCAGCTGATGAGGCAAAGAATCCTGTGattggtgcgactacagcacaa	Paired with CsaB-R to insert the El Tor TcpA EP3 epitope into CsaB
El Tor_TcpA_EP3-R	CTTTGCCTCATCAGCTGAAACCTTACCtgacgttccaaaatttaattc	Paired with CsaB-F to insert the El Tor TcpA EP3 epitope into CsaB
El Tor_TcpA_EP4-F	GCTGATCTTGGTGATTTCGAAACGAGTGTCgcgactacagcacaagcacca	Paired with CsaB-R to insert the El Tor TcpA EP4 epitope into CsaB
El Tor_TcpA_EP4-R	GAAATCACCAAGATCAGCGACAGCAGCGAAtgacgttccaaaatttaattc	Paired with CsaB-F to insert the El Tor TcpA EP4 epitope into CsaB
El Tor_TcpA_EP5-F	ACTGGCGCTGGCGTAATTAAGTCCattggtgcgactacagcacaagca	Paired with CsaB-R to insert the El Tor TcpA EP5 epitope into CsaB
El Tor_TcpA_EP5-R	AATTACGCCAGCGCCAGTAGCAGCATCTGCtgacgttccaaaatttaattc	Paired with CsaB-F to insert the El Tor TcpA EP5 epitope into CsaB
El Tor_TcpA_EP6-F	AACCTAACTAATATCACGCATGTTGAGgcgactacagcacaagcaccaacg	Paired with CsaB-R to insert the El Tor TcpA EP6 epitope into CsaB
El Tor_TcpA_EP6-R	CGTGATATTAGTTAGGTTTAAGTTGGCACTTCCtgacgttccaaaatttaa	Paired with CsaB-F to insert the El Tor TcpA EP6 epitope into CsaB
Classical_TcpA-F	CGGGCTAGCATGACATTACTCGAAGTGATCATC	Forward primer to amplify the classical TcpA, with NheI site
Classical_TcpA-R	TCACGGCCGTTATTAGCTGTTACCAAATGCAACGCCGAA	Reverse primer to amplify the classical TcpA, with EagI site
El Tor TcpA-F	CCCATGGGCATGACATTACTCGAAGTAATCATT	Forward primer to amplify the El Tor TcpA, with NcoI site
El Tor TcpA-R	TCACGGCCGTTATTAACTGTTACCAAAAGCTACTGT	Reverse primer to amplify the El Tor TcpA, with EagI site

^*a*^ Nucleotides underlined indicate a restriction enzyme site. For the primers to insert each TcpA epitope into the CsaB carrier, nucleotides in uppercase are from each TcpA epitope, and those in lowercase are of the CsaB carrier.

Additionally, the TcpA gene of O1 classical (PCR amplified from O395 genomic DNA) and O1 El Tor (PCR amplified from N16961 genomic DNA; contains an NheI restriction site) were cloned into pET28α at the NheI/EagI or NcoI/EagI sites, respectively.

### TcpA epitope fusion protein expression, purification, and characterization

Recombinant epitope fusion proteins, the full-length classical or El Tor biotype-specific TcpA, and the carrier protein CsaB, expressed by *E. coli* BL21(DE3) strain, were extracted with bacterial protein extraction reagent (B-PER) (Thermo Fisher Scientific, Rochester, NY, USA). As described previously (51), single colonies containing the individual constructs were inoculated into 10 mL of Luria-Bertani (LB) broth containing kanamycin (30 μg/mL) and incubated overnight at 37°C. Two milliliters of log-phase culture was transferred to 200 mL of fresh 2× YT medium supplemented with kanamycin (as above). The cultures were then grown at 37°C in a shaker incubator until they reached an optical density at 600 nm (OD_₆₀₀_) of approximately 0.5–0.7. Protein expression was induced with 1 mM isopropyl β-D-1-thiogalactopyranoside (Sigma-Aldrich, MO, USA), and cultures were incubated for an additional 4 hours at 37°C with shaking (200 rpm).

Bacteria were pelleted by centrifuging at 13,000 × *g* for 15 minutes at 4°C and then stored overnight at −80°C. The pellet was thawed and resuspended in 10 mL of Bacterial Protein Extraction Reagent (Thermo Fisher Scientific, Rochester, NY, USA) on ice. The suspension was sonicated several times to disrupt the bacteria and then used to extract inclusion body proteins with B-PER according to the manufacturer’s protocol.

Extracted inclusion body proteins were solubilized with solubilization buffer, 50 mM N-cyclohexyl-3-aminopropanesulfonic acid (pH 11), 0.3% N-lauroylsarcosine, and 1 mM dithiothreitol (DTT). Solubilized proteins were refolded with refolding-dialysis buffer (20 mM Tris-HCl [pH 8.5] and 0.1 mM DTT) for 3–4 hours at 4°C, followed by two to three buffer exchanges, every 8 hours, with DTT-free refolding-dialysis buffer.

Refolded TcpA epitope fusion proteins (10 μg) were examined in 15% SDS-PAGE, followed by Coomassie Brilliant Blue staining for purity and integrity assessment and Western blot using mouse anti-TcpA polyclonal antisera (1:3,000 dilution) and an IRDye-conjugated goat anti-mouse IgG secondary antibody (1:10,000 dilution; LI-COR Biosciences, Lincoln, NE, USA). Total proteins from host *E. coli* BL21 or recombinant carrier protein CsaB were also included.

### Mouse intramuscular immunization with TcpA epitope fusion protein

To assess the immunogenicity of recombinant CsaB-TcpA epitope fusion proteins to the TcpA of each biotype, female BALB/c mice (8 weeks old; five mice per group) were immunized intramuscularly with each respective CsaB-TcpA epitope fusion protein in a total of 40 µg protein (1 µg/µL), adjuvanted with 0.2 µg of double mutant heat-labile toxin (LT_R192G/L211A_, 0.1 µg/µL; supplied by PATH). The full-length recombinant classical TcpA and El Tor TcpA were included as the positive control, and PBS was used as the negative control. Mice received two booster injections of the same formulation, administered every 2 weeks after the prime. All animals were euthanized on day 42, as per protocol IACUC #23216, approved by the University of Illinois at Urbana-Champaign IACUC.

### Mouse serum anti-TcpA IgG antibody titration

Sera obtained from each mouse 2 weeks after the final booster were used to measure antigen-specific IgG responses in an indirect enzyme-linked immunosorbent assay. Immulon 2-HB 96-well plates (Thermo Fisher Scientific) were coated with 100 ng per well of the full-length classical or El Tor TcpA recombinant protein in 100 µL of 50 mM bicarbonate/carbonate coating buffer. After incubation at 37°C for 1 hour, followed by 4°C overnight, the plate wells were blocked with 1× PBS containing 0.05% Tween 20 (PBST) supplemented with 10% skim milk for 1 hour at 37°C. After three washes, the wells were incubated with twofold serial dilutions of each mouse serum for 1 hour at 37°C. The wells were rewashed with PBST and incubated with horseradish peroxidase-conjugated goat anti-mouse IgG secondary antibody (1:5,000; Bethyl Laboratories, Montgomery, TX, USA) for 1 hour at 37°C and then incubated with 3,3′,5,5′-tetramethylbenzidine substrate (KPL, Gaithersburg, MD, USA). The optical density at 650 nm (OD_650_) was measured and recorded with the SPECTROstarNano (BMG Labtech, Germany) and converted to IgG titers using the highest dilution, given an OD > 0.3 (after subtracting background readings), and then multiplied by the adjusted OD value, on a log_10_ scale.

### Mouse serum antibody adherence inhibition against *V. cholerae*

Mouse serum antibody functional activity against *V. cholerae* bacterial adherence was assessed using Caco-2 cells (ATCC #HT-37). The Caco-2 cell line has been used to study *in vitro* attachment/adherence of *V. cholerae* (20, 52, 53). Following previously described protocols (20, 47, 51, 54, 55), with minor modifications, *V. cholerae* O1 El Tor N16961, O1 classical O395, or O139 Bengal bacteria (12.5 μL, 1 × 10^6^ bacteria) were incubated with 20 µL of heat-inactivated mouse serum for 30 minutes at room temperature on a shaker set to 50 rpm. The serum-treated bacteria were then added to Caco-2 cell monolayers (95%–100% confluent; 1 × 10^5^ cells) in a 48-well plate and incubated at 37°C in a CO_₂_ incubator for 1 hour to allow adherence. After incubation, the cells were gently washed twice to remove non-adherent bacteria, dislodged, and harvested. The recovered cell suspensions containing adherent bacteria were serially diluted in PBS (300 μL) and plated onto LB agar plates. Colony-forming units were counted after overnight incubation at 37°C.

### Statistical analyses

GraphPad Prism version 10 (GraphPad Software, San Diego, CA, USA) was used to analyze the difference in antibody titers from TcpA epitope fusions and adherence inhibition activities against *Vibrio cholerae* O1 biotypes classical and El Tor, as well as O139, from mouse serum antibodies raised against each biotype-specific TcpA epitope fusion protein, using Tukey’s multiple comparisons *post hoc* test and one-way analysis of variance. A *P*-value of less than 0.05 indicated a statistically significant difference.

## Data Availability

All data are included. Original data sets are available upon request.
